# Regulation of MicroRNA-155 and Its Related Genes Expression by Inositol Hexaphosphate in Colon Cancer Cells

**DOI:** 10.3390/molecules24224153

**Published:** 2019-11-16

**Authors:** Małgorzata Kapral, Joanna Wawszczyk, Ludmiła Węglarz

**Affiliations:** Department of Biochemistry, Faculty of Pharmaceutical Sciences in Sosnowiec, Medical University of Silesia in Katowice, Jedności 8, 41-200 Sosnowiec, Poland; jwawszczyk@sum.edu.pl (J.W.); lweglarz@sum.edu.pl (L.W.)

**Keywords:** IP6, miRNAs, miR-155, FOXO3a, HIF-1α, ELK3, colon cancer

## Abstract

Inositol hexaphosphate (IP6), a natural dietary component, has been found as an antitumor agent by stimulating apoptosis and inhibiting cancer cell proliferation, their migration, and metastasis in diverse cancers including colon cancer. However, molecular mechanisms of its action have not been well understood. In recent years, microRNAs (miRNAs) have been reported to play important roles in a broad range of biologic processes, such as cell growth, proliferation, apoptosis, or autophagy. These small noncoding molecules regulate post-transcriptional expression of targets genes via degradation of transcript or inhibition of protein synthesis. Aberrant expression and/or dysregulation of miRNAs have been characterized during tumor development and progression, thus, they are potential molecular targets for cancer prevention. The aim of this study was to investigate the effect of IP6 on the miRNAs expression profile in Caco-2 colon cancer cells. 84 miRNAs were analyzed in Caco-2 cells treated with 2.5 mM and 5 mM IP6 by the use of PCR (Polymerase Chain Reaction) array. The effect of 5 mM IP6 on selected potential *miR-155* targets was determined by real-time (RT)-qPCR and ELISA (quantitative Polymerase Chain Reaction and Enzyme-Linked Immunosorbent Assay )method. The results indicated alteration in the specific 10 miRNA expression in human colon cancer cells following their treatment with 5 mM IP6. It down-regulated 8 miRNAs (*miR-155*, *miR-210*, *miR-144*, *miR-194*, *miR-26b*, *miR-126*, *miR-302c*, and *miR-29a*) and up-regulated 2 miRNAs (*miR-223* and *miR-196b*). In silico analysis revealed that *FOXO3a*, *HIF-1α*, and *ELK3* mRNAs are those of predicted targets of *miR-155*. IP6 at the concentration of 5 mM markedly induced *FOXO3a* and *HIF-1a* genes’ expression at both mRNA and protein level and decreased the amount of *ELK3* mRNA as well as protein concentration in comparison to the control. In conclusion, the present study indicates that one of the mechanisms of antitumor potential of IP6 is down-regulation of the *miR-155* expression in human colon cancer cells. Moreover, the expression of genes that are targeted by miRNA are also modulated by IP6.

## 1. Introduction

Chemoprevention is the use of natural, synthetic, or biologic chemical agents to suppress or to reverse the initial phase of carcinogenesis or to prevent carcinogenic progression to invasive cancer. Various epidemiological and preclinical studies have convincingly argued the role of some dietary agents, such as isoflavones, catechins, lycopene, or isothiocyanates, to be involved in preventing the occurrence of both solid and hematologic malignancies as well as their treatment [1,2].

Inositol hexaphosphate (InsP6, IP6) is a natural fiber-associated dietary component mainly found in cereals, legumes, nuts, oil seeds, and wheat bran. It is a derivative of myo-inositol, of which all carbon atoms are bonded to phosphate groups [3,4]. In the last years, this phytochemical has attracted increasing attention and interest due to its wide range of health-beneficial effects. In many studies, IP6 has been shown to exert antioxidant, anti-inflammatory, and anti-aging effects in many biological systems. It participates in various vital cell functions such as signal transduction, RNA export, DNA repair, energy transduction, and ATP (Adenosine 5′-triphosphate) regeneration [5]. Furthermore, IP6 lowers serum cholesterol and triglycerides [6] as well as limits the formation of kidney stones [7]. However, IP6 is best appreciated for its anti-cancer properties, resulting from its capability of reduction cell proliferation, apoptosis induction, and tumor metastasis inhibition [8,9,10]. The outcomes of many experiments have shown that IP6 broad spectrum activities involve its impact on various intracellular signaling pathways, such as PI3K/AKT (Phosphatidylinositol-3 Kinase/AKT Serine/Threonine Kinase 1) [11,12] and expression of genes encoding key cellular proteins like p53, p21, p27, BCL-2, and MMPs [13,14,15,16,17,18,19]. The anti-cancer properties of dietary IP6 have previously been demonstrated by both in vitro and in vivo experimental cancer models including, prostate, breast, bladder, and colon [10,12,13,15,16,19]. Based on the above-mentioned IP6 properties, it could be considered as a good chemopreventive and chemotherapeutic agent. However, the molecular mechanisms underlying its biological activity still need to be investigated.

Over the past decades, the scientific investigation on microRNAs (miRNAs, miRs) has indicated their promising role in cancer diagnosis, target intervention, and cancer treatments. The short noncoding RNA containing approximately 22 nt, participate in the regulation of various cellular processes such as proliferation, differentiation, apoptosis, autophagy, and physiological metabolism. Generally, miRNAs regulate gene expression by binding to the 3′-untranslated region (3′UTR) and also to the coding sequence of target mRNA, causing transcripts degradation or inhibition of their translation to functional proteins [20,21]. Currently, the existence of more than 1000 miRNAs has been estimated in human cells (comprising approximately 3% of all currently known genes in the human genome), which makes them one of the largest classes of gene expression regulators [22]. Importantly, each of the miRNAs can regulate hundreds or even thousands of target genes and, on the other hand, a single gene can be a target for multiple miRNAs [23]. The aberrant expression of miRNAs may evoke an increase in cell proliferation, invasiveness, angiogenesis, or inhibition of apoptosis, ultimately resulting in tumor formation. Therefore, they could function as oncogenes (*miR-21*, *miR-155*) or tumor suppressor genes (*let-7*, *miR-15*, *miR-16*) [22,23,24,25,26,27]. Taking this fact into account, the expression of specific miRNAs may provide valuable information for to prognostic and diagnostic purposes, and also propose them as emerging targets for cancer therapy. Many studies have shown that natural agents such as curcumin, resveratrol, isoflavone, and epigallocatechin-3-gallate could alter the expression of one or more miRNAs, leading to the modulation of their target genes’ expression and then, the inhibition of cancer cell growth, induction of apoptosis, reversal of epithelial-mesenchymal transition, or enhancement of efficacy of conventional cancer therapeutics [28,29,30,31]. 

According to the broad spectrum of IP6 action, we hypothesize that IP6 may exert its anticancer properties, in part by altering the expression of miRNAs. Thus, the purpose of this study was to determine whether inositol hexaphosphate modifies the expression profile of miRNAs in Caco-2 human colon cancer cells as well as how it affects the expression of target genes of the selected miRNA molecule.

## 2. Results 

### 2.1. Cytotoxic Effect of Inositol Hexaphosphate (IP6) on Caco-2 Cells

**Figure 1:**
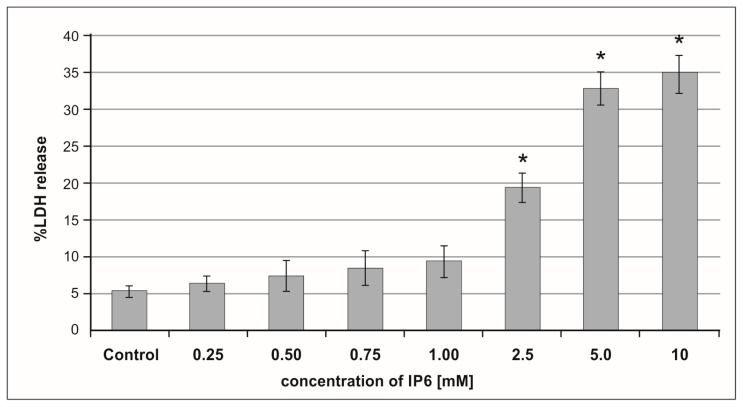
The effect of inositol hexaphosphate (IP6) on lactate dehydrogenase (LDH) release from Caco-2 cells after 48 h of incubation. The results are presented as mean ± standard deviation (SD) of six separate experiments, * *p* < 0.05 compared with untreated controls.

Lactate dehydrogenase (LDH) activity is widely used as a marker of drug or chemical cytotoxic properties. IP6-induced cell membrane damage was assessed by the LDH release assay. A dose-dependent cytotoxic effect of IP6 on Caco-2 cells was detected. LDH activity was very low in the control and cultures treated with IP6 at concentration up to 1 mM. The treatment of cells with IP6 at a concentration in the range of 2.5–10 mM resulted in an increasing leakage of LDH out of the cells (Figure 1). Caco-2 exposed to 5 mM and 10 mM IP6 exhibited a significant increase in LDH release when compared to those treated with 2.5 mM IP6 (*p* < 0.001). A similar level of LDH in cultures stimulated with both 5 mM and 10 mM IP6 was observed (*p* = 0.6645). Based on the results of this experiment, IP6 2.5 mM and 5 mM concentrations were selected in the further experiments.

### 2.2. Effect of IP6 on MicroRNA (miRNA) Expression Profile in Caco-2 Cells

**Figure 2:**
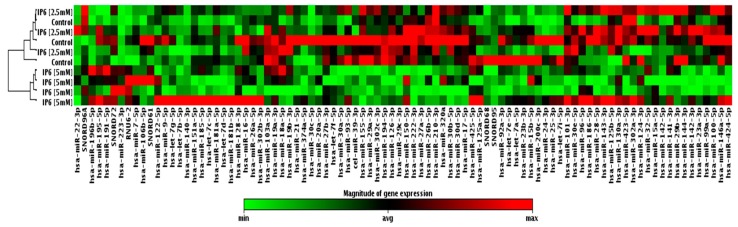
Clustergram and heatmap graph represents the expression level of 84 microRNAs (miRNAs) in untreated cells and Caco-2 treated with IP6 at concentrations of 2.5 mM and 5 mM.

**Figure 3:**
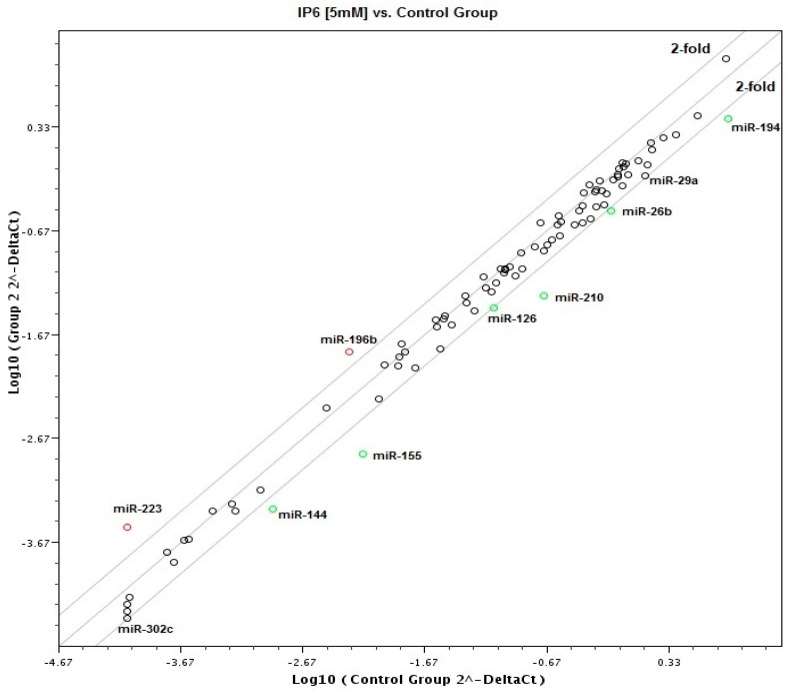
Scatter plots of miRNAs expression in untreated cells versus Caco-2 cells treated with 5 mM IP6 (x-axis represents the 2^−ΔCt^ value of control cells, and y-axis represents the 2^−ΔCt^ value of IP6-treated cells). The expression value of each human miRNA was plotted as a scatter plot, and threshold lines were drawn at a 2-fold expression change.

Taking advantage of a commercially available PCR-based assay platform for profiling expression analysis, which relies on fluorescent detection of amplified products by the SYBR Green I dye, the 84 miRNAs expression profile in Caco-2 cells treated with IP6 was determined. At first, samples taken from the control and cells treated with 2.5 mM and 5 mM IP6 have been classified based on similarities in the global miRNA expression with the use of cluster analysis, and they appeared to cluster into two distinct groups. Caco-2 treated with 5 mM IP6 clustered together, which indicates a different miRNA expression profile compared to the control and 2.5 mM IP6-stimulated cells. On the contrary to 2.5 mM IP6, its doubled concentration altered the expression of several miRNAs (Figure 2). In the next step of the analysis, miRNAs showing differential expression in Caco-2 exposed to 5 mM IP6 were identified (Figure 3). We were able to point at 8 miRNAs which were differentially expressed in the two groups. Out of 84 miRNAs screened, based on two criteria (statistical significance and at least 2-fold change), 6 miRNAs were down-regulated and 2 miRNAs were upregulated by 5 mM IP6, as compared to the untreated control (Table 1). The strongest change was observed in the case of *miR-155*, *miR-210*, and *miR-223*. The treatment of cells with 5 mM IP6 resulted in about a 4-fold decreased expression of *miR-155* and *miR-210* as compared to the control. Furthermore, two miRNAs, i.e., *miR-29a* and *miR-302c*, showed a 1.9-fold reduction in expression in response to 5 mM IP6. Simultaneously, 5 mM IP6 stimulated the expression of *miR-223* and *miR-196b* by 3.89- and 2.87-fold, respectively. 

In order to predict potential target genes for selected miRNAs, in silico analysis using TargetScan7.1 (http://www.targetscan.org/) was performed. This approach was based on the principle that miRNAs of target genes should contain miRNA’s binding sites in their 3′-UTR (3′-Untranslated Region). The overall scoring of a miRNA binding site, denoted as the context score, depends on binding features, the localization of the binding site within the 3′UTR, and the AU content of the area flanking the binding site [32]. The results of bioinformatics analysis revealed that there were 44 possible target genes for *miR-210*, 1043 possible target genes for *miR-144*, 5593 possible target genes for *miR-126*, and 1045 possible target genes for *miR-26b*. The 364 and 412 possible target genes were detected for genes *miR-196* and *miR-223*, respectively (data not shown). 

**Figure 4:**
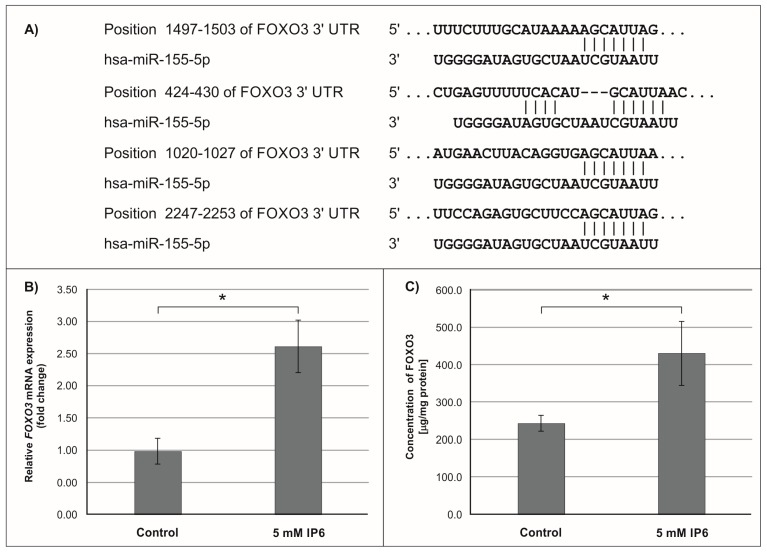
Effect of 5 mM IP6 on *FOXO3a* gene expression in Caco-2 cells at 24 h. (**A**) Schematic of the putative binding sites of *miR-155-5p* in 3′-UTR of *FOXO3* mRNA, as determined by bioinformatics analysis. (**B**) Changes in *FOXO3a* mRNA expression in Caco-2 cells, as determined by RT-qPCR. (**C**) Changes in *FOXO3a* protein concentration in Caco-2 cells, as determined by ELISA test. The results are presented as mean ± SD of three separate experiments; * *p* < 0.05 versus control.

**Figure 5:**
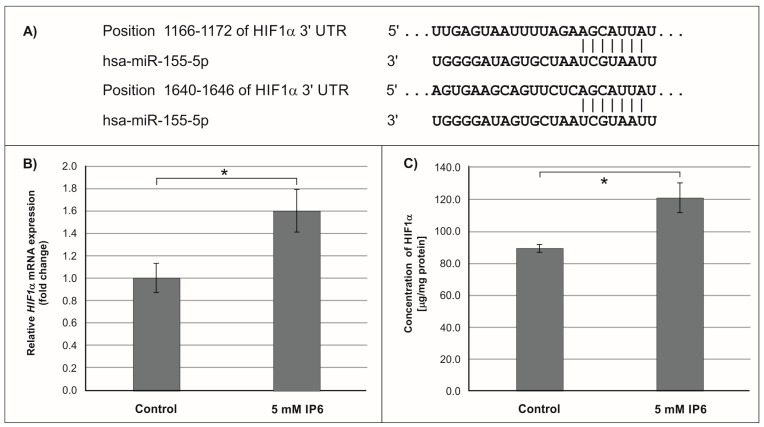
Effect of 5 mM IP6 on *HIF-1α* gene expression in Caco-2 cells at 24 h. (**A**) Schematic of the putative binding sites of *miR-155-5p* in 3′-UTR of *HIF-1α* miRNA, as determined by bioinformatics analysis. (**B**) Changes in *HIF-1α* mRNA expression in Caco-2 cells, as determined by RT-qPCR. (**C**) Changes in *HIF-1α* protein concentration in Caco-2 cells, as determined by ELISA test. The results are presented as mean ± SD of three separate experiments; * *p* < 0.05 versus control.

**Figure 6:**
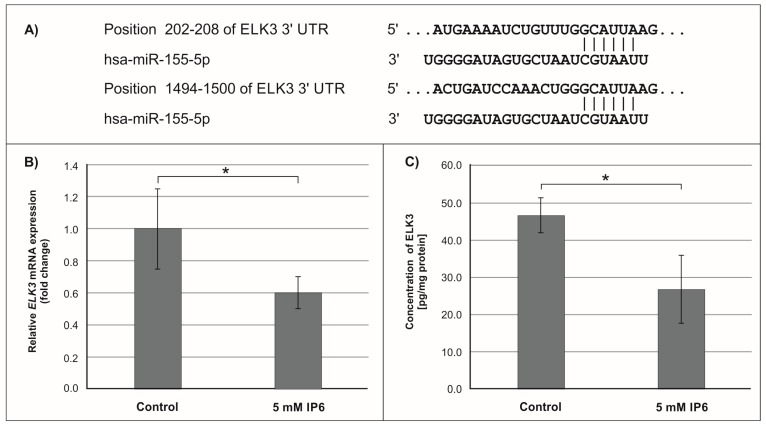
Effect of 5 mM IP6 on *ELK3* gene expression in Caco-2 cells at 24 h. (**A**) Schematic of the putative binding sites of *miR-155-5p* in 3′-UTR of *ELK3* mRNA, as determined by bioinformatics analysis. (**B**) Changes in *ELK3* mRNA expression in Caco-2 cells, as determined by RT-qPCR. (**C**) Changes in *ELK3* protein concentration in Caco-2 cells, as determined by ELISA test. The results are presented as mean ± SD of three separate experiments; * *p* < 0.05 versus control.

Subsequently, we focused on *miR-155*, the microRNA that showed the highest change in expression in Caco-2 cells treated with 5 mM IP6. In silico analysis indicated 552 genes whose 3′-UTR sequences contained tandem of predicted binding sites for *miR-155* (Figure 4A, Figure 5A and Figure 6A). The following genes: *H3F3A*, *TP53INP1*, *ETS1*, *FOS*, *FOXO3a*, *IKBKE*, *HIF-1A*, *ELK3*, *TGFBR2*, *IL6R*, *PIK3CA*, and *E2F2* have been found in this group (Table 2).

### 2.3. The Influence of IP6 on the Expression of Genes Encoding FOXO3a, HIF-1A, and ELK3 in Caco-2 Cells

In the next step of the study, the effect of 5 mM IP6 on the expression of genes potentially modulated by *miR-155* in Caco-2 cells has been evaluated. We selected three genes, i.e., *FOXO3a*, *HIF-1α,* and *ELK3* that encode transcription factors. Expression of these genes was detected at both mRNA and protein levels. The results are presented in Figure 4, Figure 5 and Figure 6. The exposure of the cells to IP6 at the concentrations of 5 mM for 24 h resulted in an up-expression of *FOXO3a* and *HIF-1a* genes at both mRNA and protein levels, as compared with untreated cells (Figure 4B,C and Figure 5B,C). On the contrary, the treatment of cells with 5 mM IP6 decreased the amount of *ELK3* mRNA as well as protein concentration in comparison to the control (Figure 6B,C).

## 3. Discussion

This study has provided significant evidence in the support of mechanisms elucidating the anticancer effect of inositol hexaphosphate. We found that IP6 can exert its biological activity via influence on the expression of some microRNAs which post-transcriptionally control the expression of related genes and consequently affect corresponding cellular processes. For the last few years, we have directed our attention to colorectal cancer (CRC), which is recognized as one of the most common malignant tumors of cancer-related deaths worldwide [33]. Among human cancers, CRC is a good candidate for chemoprevention due to the long precancerous stage before adenomas develop into cancer. Epidemiological studies have shown that consumption of a diet including fruits and vegetables is strongly associated with a reduced risk of colon cancer [34,35]. A large number of naturally occurring compounds isolated from plants have been shown to possess a possible anticancer activity [36]. The use of these agents is promising because they have minimal toxicity compared to conventional chemotherapy and they could influence multiple signaling pathways. Therefore, phytochemicals can be used alone or as an adjuvant in chemotherapy to improve therapeutic efficacy by overcoming drug resistance and reducing drug-induced toxicity [37,38]. Inositol hexaphosphate shows such characteristics. In vitro and in vivo studies demonstrated that IP6 targets cancer through multiple pathways and signaling molecules. It has been found to block protein kinases (e.g., phosphatidylinositol-3 kinase (PI3K), protein kinase C (PKC), protein kinase B (PKB) and mitogen-activated protein kinases (MAPK)), as well as to modulate transcriptional factors such as activating protein-1 (AP-1) and nuclear factor κB (NFκB) [12,16,19,39]. 

During the last several years, an increasing number of reports have documented the key role of miRNAs in the regulation of many cellular processes by controlling protein synthesis. miRNAs can act directly on the target miRNA or indirectly by regulating intermediate components, such as genes that encode transcription factors, which in turn control the expression of various genes [40]. Phuah and Nagoor [38] conclude that, as natural agents exert their anticancer effects by targeting multiple signaling pathways, and miRNAs regulate diverse biological processes including cell proliferation and programmed cell death, it is thought that miRNAs could play a role in regulating the response towards natural agents. The strategies for the regulation of miRNAs in cancer could allow the inactivation of oncogenic miRNAs, activation of tumor suppressor miRNAs, and targeting specific miRNAs to restore drug sensitivity [37]. 

The aim of the present study was to examine whether microRNAs could be involved in the response of colon cancer cells to the treatment with inositol hexaphosphate. To the best of our knowledge, the influence of IP6 on the miRNAs expression in human colon cancer cells has not been evaluated, as yet.

To determine the effect of IP6 on the miRNA profile expression in colon cancer, Caco-2 cells were incubated with 2.5 mM and 5 mM of IP6 for 24 h. As observed in this study, 2.5 mM IP6 had no influence on miRNAs expression, while 5 mM IP6 (a concentration corresponding to its physiological amount in the human large gut reaching 4 mM [41]) altered the expression of 10 miRNAs. Among them, 2 miRNAs were up-regulated (*miR-223* and *miR-196b*) and 8 were down-regulated (*miR-155*, *miR-210*, *miR-144*, *miR-194*, *miR-26b*, *miR-126*, *miR-302c*, and *miR-29a*). Importantly, *miR-155* was the most down-regulated miRNA in cells exposed to IP6. Therefore, in the next step of the study, we focused on *miR-155* and evaluated how IP6 affected three selected target transcripts of this molecule (*FOXO3a*, *HIF-1α*, and *ELK3*). 

The *miR-155* is recognized as a multifunctional microRNA. Since it is known to target many transcripts including those encoding transcription factors, cytokines, and enzymes, it may be involved in the control of several cellular processes, such as cell growth and survival, cell migration and invasion, cell adhesion junction, apoptosis, and proliferation [42,43,44]. Consequently, this small molecule plays a pivotal role as an oncogenic miRNA in tumorigenesis and cancer progression. The results of many studies showed an increased expression of *miR-155* in various cancers, including colon cancer [25,44,45,46]. An elevated *miR-155* level was accompanied by down-regulation of its target gene transcripts and resulted in inhibition of translation process. Liu et al. [45] observed that upregulating the expression of *miR-155* in SW-480 colorectal cancer cells promoted distant invasion and metastasis of tumor cells through activating the Wnt/β-catenin signal pathway. Additionally, it has been shown to be involved in TGF-β/SMAD-induced epithelial-mesenchymal transition (EMT) [47]. Moreover, a number of target genes of *miR-155* affect other cancer-related processes, such as cell growth and survival (*CCND1* and *GAB3*), cell adhesion junction (*ANKRD6* and *SMAD2*), proliferation (*SOCS1* and *STAT3*), and apoptosis (*TP53BP1*) [42,48]. The published data also suggested that *miR-155* may influence inflammation-induced development of cancer [29,49,50]. Recently, O’Connell and co-workers [51] reported that *miR-155* targets inositol phosphatase (SHIP), the enzyme that cleaves phosphatidylinositol (3,4,5)-trisphosphate (PIP_3_) to generate phosphatidylinositol 3,4-bisphosphate (PI 3,4-P_2_), i.e., the factor implicated in the regulation of kinase AKT activity. 

Furthermore, *miR-155* exerts its oncogenic function by targeting critical suppressor genes, such as *FOXO3a*. *FOXO3a* (*FOXO3*) is a member of the forkhead box class O (FOXO) subfamily of transcription factors that mediate a variety of cellular processes including apoptosis, proliferation, cell cycle progression, and DNA damage. *FOXO3* induces cell cycle arrest mediated through transcription of multiple cell cycle kinase inhibitors (CKI), such as p27 (CDKN1B) and p21 (CDKN1A). It is also involved in the regulation of autophagy in cancer cells. *FOXO3a* acts as a tumor suppressor and it is frequently inactivated in cancer cells, which is followed by the initiation and progression of cancer [52,53]. *FOXO3a* is directly targeted by *miR-155,* which blocks translation of this factor. Experimental studies revealed that the overexpression of *miR-155* down-regulates the expression of *FOXO3a* protein. Indeed, Gao et al. [54] revealed that overexpression of *miR-155* in colon cancer tissues and human colon cancer cell lines (HT29 and SW620) was associated with decreased levels of *FOXO3a*. In addition, their study showed that *miR-155* may not only promote colon cancer growth but also increase colon cancer chemo-resistance to cisplatin through inhibiting *FOXO3a* expression. So, reduction of *miR-155* expression is a potential interest of the search for chemopreventive and chemotherapeutical agents. The findings of this study revealed that IP6 decreased the expression of *miR-155* in colon cancer cells, which was accompanied by up-regulated expression of the *FOXO3a* gene at both miRNA and protein levels. 

Hypoxia-inducible factor 1α (*HIF-1α*) is one of the subunits of hypoxia-inducible factor 1 (*HIF-1*) [55]. *HIF-1* is an oxygen-dependent transcriptional activator. More than 60 putative *HIF-1* target genes have been identified. The target genes of *HIF-1* are especially related to angiogenesis and cell proliferation/survival. *HIF-1* regulates the expression of all enzymes of the glycolytic pathway, as well as the expression of the glucose transporters GLUT1 and GLUT3, which mediate cellular glucose uptake. *HIF-1α* also plays a role in many diseases, such as cancer, that generate a hypoxic microenvironment [55,56]. The in vitro studies by Robertson et al. [57] revealed that *miR-155-5p* specifically down-regulated *HIF-1α*. The results of our study demonstrated that in Caco-2 cells treated with 5 mM IP6, the amount of both transcript and protein of *HIF-1α* slightly increased in comparison to the control. This may suggest that such an effect was caused by IP6-reduced *miR-155* expression.

The oncogenic *miR-155* can also control the expression of *ELK3* miRNA, of which two conserved *miR-155-5p* target sites in the 3′-UTR of *ELK3* have been identified [57]. *ELK3* belongs to the ETS transcriptional factors family (domain-containing protein ETS) and it participates in MAPK kinase signaling. It represses the transcription of the protooncogene c-Fos. Overexpression of *ELK3* has been shown to inhibit proliferation of cancer cells. This factor is also involved in the hypoxia response, angiogenesis, and vascular integrity. Although *ELK3* functions as a transcriptional repressor, it can be transformed into a transcriptional activator via RAS/ERK signaling [57,58,59]. Earlier published data have shown that, in vein endothelial (HUVEC) cells *ELK3,* miRNA was down-regulated by *miR-155,* which also significantly decreased *ELK3* protein levels, whereas the *miR-155* inhibitor increased *ELK3* levels to a small extent [57]. Our study demonstrated that both *ELK3* miRNA and protein amounts were decreased in IP6-treated cells, in which *miR-155* was down-regulated. Therefore, we can conclude that this change in *ELK3* gene expression was independent of the regulatory effect of IP6 on *miR-155*. Perhaps, in Caco-2 colon cancer cells, other specific microRNAs may be able to regulate *ELK3* miRNA expression at the post-transcriptional level.

In our previous studies, we analyzed the effect of IP6 on Caco-2 colon cancer cells proliferation and apoptosis. We have shown that IP6 reduce proliferation and induce apoptosis of Caco-2 through the inhibition of the AKT/mTOR pathway and the mTOR effector, followed by modulation of the expression and activity of several key components of this pathway. Furthermore, IP6 regulated the expression of cyclin D1 and increased the level of p21 protein, cyclin-dependent kinase inhibitor, in Caco-2 cells. Concomitantly, IP6 induced apoptosis of colon cancer cells, which was accompanied by upregulated expression of caspase 9 and caspase 3 at the transcriptional level as well as an increase in caspase-3 activity [19,60]. Numerous studies have revealed that transcriptional factor *FOXO3* regulates the expression of genes encoding p21, p27, cyclin D1, and caspases. On the other hand, *miRNA-155* negatively regulates the expression of *FOXO3*. The findings described herein indicate that IP6 at the concertation of 5 mM alters specific miRNAs’ expression in Caco-2 cells. To the best of our knowledge, the effect of IP6 on the miRNA’s expression in human colon cancer cells is demonstrated for the first time. Furthermore, gene expression that is targeted by these IP6-regulated specific miRNAs was also modulated by this compound. Hence, it can be suggested that antitumor activity of IP6 may be due to its effect on miRNA-related changes in cancer cells.

In conclusion, the present study illustrates, for the first time, that IP6 exerts its biological functions by down-regulating the *miR-155* expression in human colon cancer cells. Further studies are warranted to investigate the interaction between IP6 and other differently expressed miRNAs, and their target miRNAs, which may provide new perspectives on understanding the anticancer and chemopreventive effect of inositol hexaphosphate.

## 4. Materials and Methods

### 4.1. Cell Culture

The human colon cancer cell line Caco-2 was purchased from American Type Tissue Collection (ATCC) (Rockville, MD, USA). The cells were grown routinely in RPMI 1640 medium (Sigma–Aldrich, St. Louis, MO, USA) supplemented with 10% fetal bovine serum (BioWest, Nuaillé, France), 100 U/mL penicillin, 100 μg/mL streptomycin (Sigma Aldrich), and 10 mM HEPES (Sigma Aldrich) in a humidified atmosphere of 5% CO_2_ at 37 °C. Cells were treated with IP6 and proceeded for analysis, as described below. 

### 4.2. Inositol Hexaphosphate (IP6)

IP6 (dipotassium salt) was purchased from Sigma–Aldrich. IP6 was dissolved in distilled water to make a 250 mM stock solution and adjusted to pH 7.4. Subsequently, it was diluted in tissue culture media to the desired concentrations of 0.25, 0.5, 0.75, 1, 2.5, 5, and 10 mM. 

### 4.3. Lactate Dehydrogenase (LDH) Assay

Leaky membranes of damaged or dead cells release the lactate dehydrogenase (LDH) into the culture medium. Increased LDH activity in the supernatants of cell cultures correlates with the percentage of dead cells. The cytotoxic effect of IP6 was analyzed by LDH release into the culture medium with the use of an In Vitro Toxicology Assay Kit, Lactate Dehydrogenase-Based (Sigma–Aldrich). The assay was performed according to the manufacturer’s protocol. Cells were seeded in 96-well plates at an initial density of 1 × 10^4^ cells per well in 200 µL culture medium and incubated for 24 h. Afterwards, the medium was aspirated and cells were exposed to the freshly prepared media containing various concentrations of IP6 (0.25–10 µM). After 48 h, LDH activity was determined both in the culture medium (extracellular LDH activity) and in the cell lysates (total LDH activity). The absorbance was measured at 492 nm and 690 nm (reference wavelength) using the Labtech LT-5000 microplate reader (Uckfield, UK). The results are shown as a percentage of total LDH released into the medium. Subsequently, the percentage of liberated enzyme was calculated for control cells and cells treated with IP6 using the following equation: LDH release (%) = extracellular LDH/total LDH × 100%. The experiment was repeated six times.

### 4.4. microRNA Isolation and microRNA Polymerase Chain Reaction Arrays

For miRNA expression studies, Caco-2 cells were seeded into six-well plates (Nunc International, Rochester, NY, USA) at a density of 4.5 × 10^5^ per well and allowed to grow to confluency in 3 mL of medium. After 3 days, the culture media were changed to media with 2% FBS (Fetal bovine serum) and cells were then cultured for 2 days. Afterwards, cells were treated with 2.5 and 5 mM IP6 for 24 h. The untreated Caco-2 cells were used as the control. Small RNAs were extracted from the control and IP6-treated cells by the use the QIAzol and miRNeasy Mini Kit (Qiagen Inc., Valencia, CA, USA) following the manufacturer’s protocol. The concentration of RNA samples was determined spectrophotometrically on the basis of absorbance values at a wavelength of 260 using a Shimadzu UV-1800 spectrophotometer (Shimadzu, Kyoto, Japan). RNA purity was judged by the ratio of absorbances at 260 and 280 nm (A260/A280) (ratios between 1.9 and 2.1 were acceptable). For each reaction, 25 ng of RNA, extracted from control and cells treated with 2.5 and 5 mM IP6, was submitted to reverse transcription with miScript II RT Kit (Qiagen) following the manufacturer’s instructions (Qiagen). The reverse transcription (RT) reaction was carried out at 37 °C for 60 min, and then the samples were heated at 95 °C for 5 min to inactivate the reverse transcriptase. To measure miRNAs, cDNA was diluted in RNase-free water. The profile expression of the 84 most abundantly expressed and best characterized miRNAs in miRBase was performed using the Human miFinder miScript miRNA PCR Array (MIHS-001Z) and miScript SYBR Green PCR Kit (Qiagen), according to the manufacturer’s protocol. Real-time qPCR analysis was done on a CFX Connect Real-Time PCR Detection System (Bio-Rad, Hercules, CA, USA) under the following conditions: 95 °C for 15 min and 40 cycles at 94 °C for 15 s, 55 °C for 30 s, 70 °C for 30 s. Specificity of PCR reaction was confirmed by determining the temperature of melting for all amplimers. The relative amount of each miRNA in the PCR array analysis was normalized to an average of four small nuclear housekeeping genes (*SNORD 61*, *SNORD 68*, *SNORD 72*, and *SNORD 96A*). The fold-change for each miRNA in IP6-treated cells relative to control cells was calculated using the 2^−ΔΔCT^ method. miRNAs were considered as potentially differing if *p* < 0.05 and at least a 2-fold change in the mean expression level between cells treated with IP6 and controls was found. The results were collected from three or four independent experiments.

### 4.5. Total RNA Extraction and Quantitative Real-Time RT-PCR (RT-qPCR)

To evaluate transcriptional activity of *FOXO3a, HIF-1α*, and *ELK3* genes, the cells were seeded at a density of 8 × 10^5^ onto 21.5 cm^2^ culture dishes (Nunc International). Then, IP6 at the concentration of 5 mM was added to cell cultures for 24 h. Total RNA was extracted from the cells with the use of TRI REAGENT (Zymo Research, Irvine, CA, USA). Detection of the expression of examined genes was carried out using a RT-qPCR technique with a SYBR Green chemistry (SYBR Green Quantitect RT-PCR Kit) (Qiagen), as was described previously [19]. Primers for *FOXO3a*, *HIF-1α*, and *ELK3* mRNAs were commercially available (Sigma–Aldrich). The expression levels of all genes in cultured cells were expressed as the fold change (FC) relative to the corresponding controls. FC values greater than one indicate a positive or an up-regulation. FC values less than one indicate a negative or down-regulation.

### 4.6. Measurement of FOXO3A, HIF-1α, and ELK3 Proteins

Expression of *FOXO3a, HIF-1α,* and *ELK3* proteins in Caco-2 cells was determined by commercially available enzyme-linked immunosorbent assay (ELISA) kits (Cloud-Clone Corp. Houston, TX, USA and Cusabio Houston, TX, USA). Cells were plated onto 100 mm dishes at a density of 3 × 10^6^ cells. After two days, the media were aspirated and cells were incubated with IP6 at the concentration of 5 mM for 24 h. Subsequently, cells were lysed in commercially available lysis buffer. The concentrations of *FOXO3A, HIF-1α* and *ELK3* proteins in cell lysates were measured with ELISA kits following the manufacturers’ protocols. Results are normalized to total cellular protein content, which was determined by the Bradford’s method (Sigma–Aldrich). 

### 4.7. Statistical Analyses

Statistical analysis was performed with the use of Statistica PL 12.0 software. All data expressed as means ± standard deviation (SD) were representative of at least three independent experiments. Comparison of more than two datasets was performed by one-way analysis of variance (ANOVA) followed by a post-hoc Tukey test. Comparison of two datasets was performed by an unpaired t-test. The significance level was assumed for *p* < 0.05. PCR array analysis of miRNA expression was performed using SABiosciences’ web-based software (http://pcrdataanalysis.sabiosciences.com/mirna.). 

## Figures and Tables

**Table 1:** MicroRNA expression changes in Caco-2 cells treated with 5 mM IP6 as compared with the control, as deduced from microRNA microarray analysis. Changes were considered significant for *p* < 0.05.

miRNA	Down/Up-Expression	Fold Change	*p*-Value
*miR-155*	down-expression	−4.32	0.0013
*miR-210*	down-expression	−3.97	0.0025
*miR-144*	down-expression	−2.71	0.049
*miR-194*	down-expression	−2.54	0.0109
*miR-26b*	down-expression	−2.17	0.0377
*miR-126*	down-expression	−2.03	0.0135
*miR-302c*	down-expression	−1.93	0.011
*miR-29a*	down-expression	−1.9	0.0197
*miR-196b*	up-expression	2.87	0.0187
*miR-223*	up-expression	3.89	0.048

**Table 2:** Selected predicted targets of *miR-155-5p*, as determined by TargetScan7.1.

Target Gene	Gene Name	Number of Conserved Sites	Number of Poorly Conserved Sites
*ZNF385D*	zinc finger protein 385D	1	4
*H3F3A*	H3 histone, family 3A	1	0
*ETS1*	v-ets avian erythroblastosis virus E26 oncogene homolog 1	2	0
*FOS*	FBJ murine osteosarcoma viral oncogene homolog	1	0
*TCF4*	transcription factor 4	3	1
*TP53INP1*	tumor protein p53 inducible nuclear protein 1	1	0
*MAP3K10*	mitogen-activated protein kinase kinase kinase 10	1	0
*SOCS1*	suppressor of cytokine signaling 1	1	0
*SMAD1*	SMAD family member 1	1	0
*KRAS*	Kirsten rat sarcoma viral oncogene homolog	1	1
*FOXO3a*	forkhead box O3a	1	3
*IKBKE*	inhibitor of kappa light polypeptide gene enhancer in B-cells, kinase epsilon	2	0
*ELK3*	ELK3, ETS-domain protein (SRF accessory protein 2)	1	1
*E2F2*	E2F transcription factor 2	2	0
*RELA*	v-rel avian reticuloendotheliosis viral oncogene homolog A	1	0
*PIK3CA*	phosphatidylinositol-4,5-bisphosphate 3-kinase, catalytic subunit alpha	1	0
*HIF1A*	hypoxia inducible factor 1, alpha subunit (basic helix-loop-helix transcription factor)	1	1
*SMAD5*	SMAD family member 5	1	1
*IL6R*	interleukin 6 receptor	1	0
*ELF4*	E74-like factor 4 (ets domain transcription factor)	1	0
*TGFBR2*	transforming growth factor, beta receptor II (70/80kDa)	1	0
*IRF2BP2*	interferon regulatory factor 2 binding protein 2	1	0
*ADAM10*	ADAM metallopeptidase domain 10	1	0
*GSK3B*	glycogen synthase kinase 3 beta	1	0
*SMAD2*	SMAD family member 2	1	1
